# An Artificial Intelligence-Based Automatic Classifier for the Presence of False Lumen Thrombosis After Frozen Elephant Trunk Operation

**DOI:** 10.3390/diagnostics14242853

**Published:** 2024-12-18

**Authors:** Anja Osswald, Konstantinos Tsagakis, Matthias Thielmann, Alan B. Lumsden, Arjang Ruhparwar, Christof Karmonik

**Affiliations:** 1 Department of Thoracic and Cardiovascular Surgery, West-German Heart and Vascular Centre, University Duisburg-Essen, 45122 Essen, Germany; konstantinos.tsagakis@uk-koeln.de (K.T.); matthias.thielmann@uk-essen.de (M.T.); ruhparwar.arjang@mh-hannover.de (A.R.); 2 Department of Vascular Surgery, Houston Methodist DeBakey Heart & Vascular Center, Houston, TX 77030, USA; ablumsden@houstonmethodist.org; 3 Translational Imaging Centre, Houston Methodist Research Institute, Houston, TX 77030, USA; ckarmonik@houstonmethodist.org

**Keywords:** aortic dissection, false-lumen thrombosis, artificial intelligence, variational autoencoders, automatic classification

## Abstract

Objective: To develop an unsupervised artificial intelligence algorithm for identifying and quantifying the presence of false lumen thrombosis (FL) after Frozen Elephant Trunk (FET) operation in computed tomography angiographic (CTA) images in an interdisciplinary approach. Methods: CTA datasets were retrospectively collected from eight patients after FET operation for aortic dissection from a single center. Of those, five patients had a residual aortic dissection with partial false lumen thrombosis, and three patients had no false lumen or thrombosis. Centerlines of the aortic lumen were defined, and images were calculated perpendicular to the centerline. Lumen and thrombosis were outlined and used as input for a variational autoencoder (VAE) using 2D convolutional neural networks (2D CNN). A 2D latent space was chosen to separate images containing false lumen patency, false lumen thrombosis and no presence of false lumen. Classified images were assigned a thrombus score for the presence or absence of FL thrombosis and an average score for each patient. Results: Images reconstructed by the trained 2D CNN VAE corresponded well to original images with thrombosis. Average thrombus scores for the five patients ranged from 0.05 to 0.36 where the highest thrombus scores coincided with the location of the largest thrombus lesion. In the three patients without large thrombus lesions, average thrombus scores ranged from 0.002 to 0.01. Conclusions: The presence and absence of a FL thrombus can be automatically classified by the 2D CNN VAE for patient-specific CTA image datasets. As FL thrombosis is an indication for positive aortic remodeling, evaluation of FL status is essential in follow-up examinations. The presented proof-of-concept is promising for the automated classification and quantification of FL thrombosis.

## 1. Introduction

Frozen Elephant Trunk (FET) operation is a hybrid procedure that allows for a single-stage treatment of complex aortic pathologies, including aortic dissections. This approach combines the surgical replacement of the aortic arch and simultaneous stent grafting of the descending aorta [1,2]. FET aims for the exclusion of entry tears and false lumen (FL) in aortic dissection, redirecting the blood flow into the true lumen. This exclusion from antegrade flow consequently results in thrombosis of the FL, a pathological space formed between the intimal and medial layers of the aortic wall during dissection. When the FL is fully thrombosed, the aortic wall stabilizes, reducing the risk of aneurysmal dilatation, rupture, and further dissection [3]. The desirable result of FL thrombosis can also lead to FL shrinkage and obliteration, favoring positive aortic remodeling, which is defined by a total aortic diameter decrease or an increase in the true lumen (TL) size while maintaining a stable total aortic diameter [4,5]. In contrast, a patent or partially thrombosed FL or an increase in FL diameter is associated with continued hemodynamic stress, progressive dilatation of the aorta, and a higher likelihood of reintervention or mortality [6].

The FL after aortic dissection can be classified into four categories:

1. Obliteration: Complete closure of the FL with no detectable lumen remaining.
2. Fully Thrombosed FL: The FL is entirely filled with thrombus, with no residual blood flow.
3. Partially Thrombosed FL: The FL contains both thrombus and residual blood flow, indicating incomplete occlusion.
4. Patent FL: No thrombus is present; the FL remains open with continuous blood flow.

The standard of care includes serial follow-up computed tomography angiographic (CTA) imaging. Traditionally, the classification of FL thrombosis has relied on manual analysis by radiologists or cardiac surgeons, which can be time-consuming and subject to inter-observer variability.

Computational algorithms for identifying and quantifying the presence of FL thrombosis may be advantageous for aiding in the medical diagnosis by providing quantitative assessment of the medical image data. The recently developed artificial intelligence (AI) algorithms, those specifically applying neural networks to patient data, provide a new powerful set of technologies, which are able to classify complex data interrelationships [7,8,9,10,11]. In particular for medical imaging data, AI-based computer-assisted support has been demonstrated to be of great interest, as the image data content can be analyzed and quantified automatically or with only minimal user interaction [12]. Traditionally, convolutional neural networks perform classifications by using image datasets that are manually annotated, which requires effort for the curation of this data. Alternatively, unsupervised learning algorithms offer a promising solution, as these algorithms can discover hidden patterns and structures in data without the need for labeled training data. One of these algorithms, the variational autoencoder (VAE), condenses information into the so-called latent space with a considerably reduced dimension [10,13,14,15,16,17]. To process two-dimensional image data, this latent space uses the output of a 2D convolutional network as input and serves as the input of another 2D convolutional network, which recreates the original images. 

Convolutional neural networks (CNNs) are a class of deep learning algorithms designed specifically for analyzing visual data. These networks mimic the human brain’s way of processing visual information by applying convolutional layers to extract hierarchical patterns, such as edges, textures, and shapes, which are crucial for image classification tasks. In medical imaging, CNNs have proven highly effective for automatically identifying and segmenting anatomical features and pathologies [18]. Leveraging these capabilities, this study employs a 2D CNN as part of a VAE to process cross-sectional CTA images of the aorta, enabling automated classification of FL after FET operation.

The aim of this proof-of-concept study was to develop an unsupervised learning artificial intelligence algorithm using variational autoencoders for the automatic classification of FL thrombosis in CTA images of patients after Frozen Elephant Trunk operation in an interdisciplinary approach consisting of clinicians and experts in imaging and computer science.

## 2. Materials and Methods

### 2.1. Patient Image Data

This study was approved by the ethics committee of the University of Duisburg-Essen (23-11102-BO), and individual patient consent was waived. For this single-center study, CTA image datasets were retrospectively obtained from eight patients. All patients received a FET operation due to either an acute or chronic aortic dissection. In five of these patients, a residual aortic dissection remained with partial thrombosis of the false lumen. Three patients did not have a residual aortic dissection after FET, and no thrombus was present. Thrombosis was characterized by the absence of contrast enhancement. The FL status was defined as thrombosed, partially thrombosed, or patent. 

For proper training of the VAE algorithm, a large number of images is necessary. In the case of CTA datasets, each individual image can be used as an input for the VAE algorithm. Therefore, a sufficiently large number of training images can be obtained even from a small number of patient datasets; the CTA scans from 8 patients resulted in a dataset of 3938 images for algorithm training. Images contained all classes of interest that were intended to be distinguished, such as patent aortic lumen, patient true/false aortic lumen, and patent aortic lumen with thrombosed FL. Through classification and scoring of these images for each patient dataset, a unique score was then attributed to each patient dataset, which may be of value in the clinical evaluation.

### 2.2. Image Pre-Processing

A centerline in the patient aortic lumen was created by manually defining a curved centerline using the 3 orthogonal MPR planes (sagittal, coronal, axial) with the 3D curved MPR tool in Horos (Horos Project, Annapolis, MD, USA; version 4.0.0 RC5). Images were then reconstructed perpendicular to the defined centerline so that the patent aortic lumen was centered in these images with the smallest possible diameter. 

The aortic lumen and the surrounding thrombus were segmented semi-manually using the Magic Wand tool (Fiji/ImageJ2 version 2.9.0/1.53t [19]) in the curved 3D MPR reconstruction, where the aorta and the centerline were straightened. After segmentation, images perpendicular from the curved 3D MPR were re-reconstructed. Segmented images were auto-centered, and the image size was recentered to 64 × 64, yielding the images for the VAE.

### 2.3. Variational Autoencoder

A Variational Autoencoder (VAE) is a type of deep generative model that learns to encode input data into a compressed latent space while preserving its essential features, allowing for probabilistic data reconstruction and generation [20,21,22].

Convolutional Neural Networks: As input into the latent space and as output to recreate the original images from the values in the latent space, two identical convolutional neural networks were created [18]. The VAE encoder CNN condenses high-dimensional input images into a structured latent space of reduced dimensionality, where relevant patterns like thrombosis or lumen patency are represented. The decoder CNN then reconstructs images from this latent space, ensuring minimal reconstruction error. From these, the representation of each image is calculated by a point (location) in feature space for that image. The VAE was implemented with tensorflow (version 2.7.0) [23] using Python (version 3.9.9) with a virtual environment created with conda (version 4.11.0). A total of 100 epochs were used to train the VAE.

**Figure 1:**
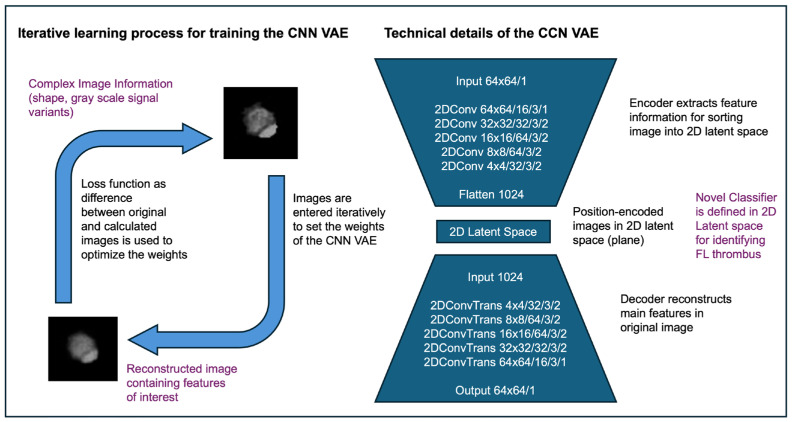
Schematic representation of the Variational Autoencoder (VAE) algorithm, consisting of an encoder and decoder with Conv2D (Convolutional two-dimensional) layer and 2DConvTrans (Transpose two-dimensional Convolutional) layers, respectively. The encoder compresses input images into a 2D latent space to classify false lumen characteristics, while the decoder reconstructs the images by upsampling this representation. Layer names, sizes, depths, and kernel/stride configurations are shown to illustrate the dimensional transformations throughout the process.

Latent Space Classification: Representative images were visualized with predicted latent space points using the decoder 2D CNN and displayed together with the distribution of the points. A normalized standard Gaussian probability distribution with a mean zero and standard deviation was used. Subspaces in the latent space of interest are identified and defined by latent space coordinates [24,25]. For this study, the latent space was divided into two subspaces separated by a straight line at location x0. Values of the x-component of the latent space smaller than x0 represent images with thrombus, values of x larger than x0 those without thrombus (Figure 1). Training of the VAE algorithm is illustrated in Video S1. This CNN-based approach effectively captured the nuances of FL thrombosis and facilitated automated scoring by mapping each image into latent space coordinates.

Score Calculation for each Dataset: Classification for each image representing an axial cross-section of the aorta was identified by the corresponding location of this image in the latent space [24,25]. Here, the thrombus score for each image was calculated as ts = (x − x0)/(xmin − x0) for images within the thrombus subspace and ts = 0 otherwise. Averaging the scores for all classified images from one dataset yielded the total thrombus score for that dataset.

## 3. Results

### 3.1. Image Pre-Processing Evaluation

**Figure 2:**
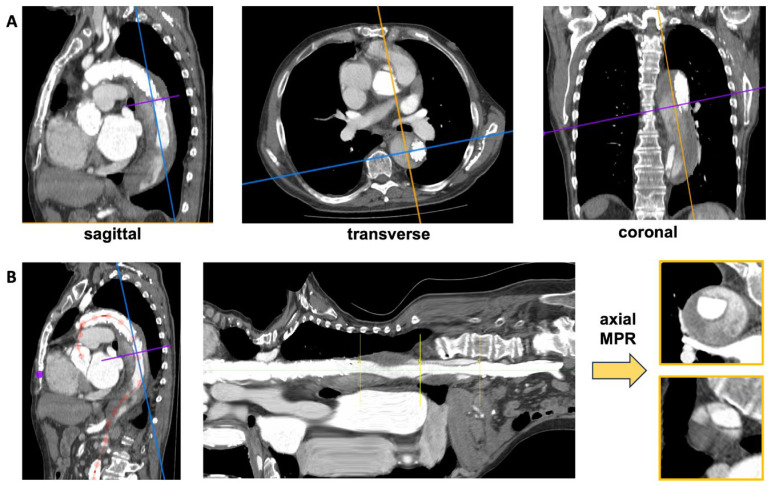
(**A**): Conventional three-plane visualization of the aorta. (**B**): On the left, the centerline is defined by red points and the line inside the aortic lumen; in the center, a 3D curved MPR reconstruction with the centerline and, consequently, the aortic lumen straightened. The perpendicular MPR slices (shown on the right) provide cross-sectional views of the aortic lumen.

The curved MPR images along the aortic center line yielded a straight presentation of the aorta and surrounding thrombus (Figure 2A). Semi-automated segmentation resulted in aortic cross-sections perpendicular to the aorta of high quality (Figure 2B), where the aortic lumen was hyperintense with thrombus being isointense relative to surrounding tissue. The septum of the aortic dissection was clearly visible. In patients with FL thrombus and patent FL, the diameter was larger than in patients without residual aortic dissections.

### 3.2. Variation Autoencoder Training

**Figure 3:**
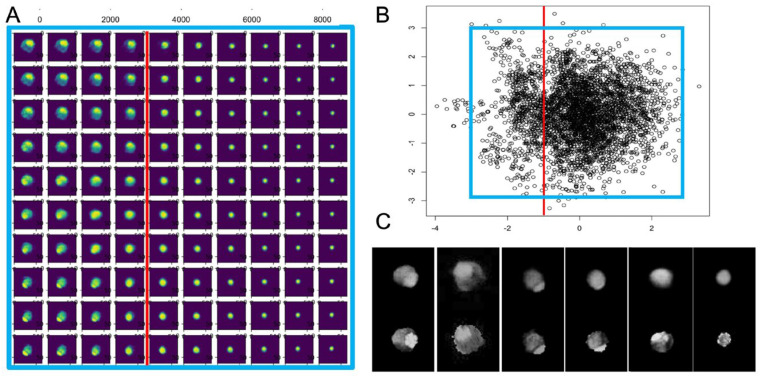
(**A**) Latent space visualization of all images used to train the VAE, with areas for reconstructed images outlined in blue. Images are classified based on aortic area and thrombus content, with the red line dividing the thrombus (left) from no thrombus (right). In the upper left are the images with a large aortic area and a large amount of thrombus, and at the bottom on the right are the ones with no thrombus and a small aortic area. (**B**) Reconstructed images from the VAE decoder mapped to the latent space ranging from −3 to 3, with the red line separating thrombus regions. (**C**) Sample comparisons of original (bottom) and reconstructed (top) images showing the true lumen with the thrombus (left), true and false lumens (middle), and only the true lumen (right).

The total number of images to train the VAE were 3938 (subject 1: 323 images; 2: 515, 3: 526, 4: 446, 5: 483, 6: 537, 7: 583, 8: 525). Convergence of the VAE was fast for all 100 epochs. The latent space generated by the encoder exhibited a bipolar distribution (Figure 3A), with distinct clusters separating images containing the thrombus from those without. This separation was achieved using a straight line at x0 = −0.8 in the latent space, where images with x-values smaller than x0 corresponded to the thrombus, while those with x-values larger than x0 represented no thrombus (Figure 3B). Images were classified based on the aortic area and thrombus amount. Good agreement between the original images (input for the VAE encoder) and the reconstructed images (output from the VAE decoder) was generally found (Figure 3C). The agreement appeared best for images containing patent true lumen together with the thrombus. The presence of patent in both true lumen and false lumen appeared in the reconstructed smaller images, as the variation of intensity with higher intensity in the true lumen corresponded to stronger flow.

### 3.3. Clinical Assessment

**Figure 4:**
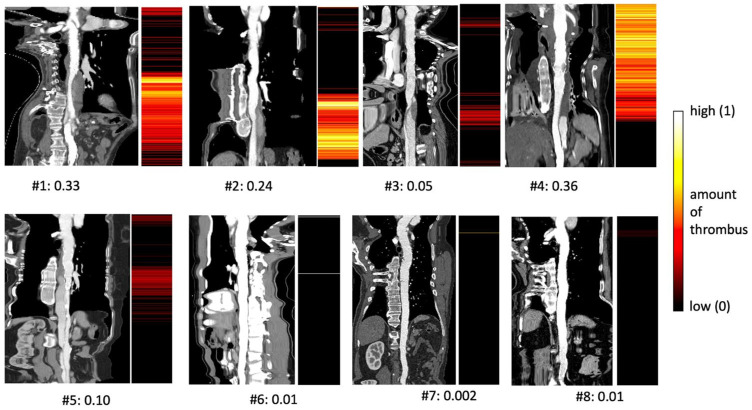
For each subject, the 3D MPR with the straight aortic lumen is shown together with a panel that contains the thrombus score in pseudo color derived from the classification for each slide perpendicular to the aortic lumen (legend on right). The total thrombus score for each subject is shown below each figure.

High amounts of thrombus were correctly identified in the aortic arch and SG area in #4 and in the downstream aorta in #1 and #2 (Figure 4). In the case of patent FL, as seen in the abdominal aorta in #3 and #5, agreeable results were achieved, showing low amounts of thrombus. Partial thrombosis resulted in red color coding, which indicates a low amount of thrombogenic material. In control cases #6–8, with a completely patent lumen and no residual dissection, no thrombus was detected by the algorithm.

### 3.4. Calculation of Thrombus Score

For all subjects, the average thrombus scores were as follows: Subject #1: 0.33, #2: 0.24, #3: 0.05, #4: 0.36, #5: 0.10, #6: 0.01, #7: 0.002, and #8: 0.01. For subjects #1–5, images classified containing thrombus corresponded well to large regions of thrombus visible in the 3D curved MPR display. In subjects #6–8, no aortic thrombus was present, which corresponded to the overall absence of thrombus classification.

## 4. Discussion

This study presents a novel approach using an unsupervised AI algorithm for the identification and quantification of FL thrombosis in patients who have undergone a FET operation for aortic dissection. The AI model, specifically a VAE based on 2D CNN, demonstrated high concordance between the reconstructed images and the original CTA images, accurately identifying and scoring the presence of FL thrombosis.

After FET, FL thrombosis is expected in the majority of patients in the thoracic aorta, and in the abdominal aorta, FL often remains patent or partially patent [26]. As FL thrombosis is an indication for positive aortic remodeling, the evaluation of FL status is essential in follow-up examinations. Currently, aortic diameters are manually measured in 2D CTA images in order to make recommendations on further treatment and time intervals of follow-up CTAs. This is a labor-intensive procedure, carried out by the interventional specialist. Providing computer assistance, e.g., by an algorithm that would allow for overall quantification of the FL, may result in reducing image processing time and would result in an advantage over only reporting mean aortic diameter changes, as they allow for positive aortic remodeling in one segment but potentially masking the overall growth. Furthermore, it would be favorable to specify with the percentage of FL, compared to the aortic diameter and TL, with a dedicated AI algorithm. The automatic classification of FL thrombosis in patients undergoing FET surgery using an unsupervised learning algorithm represents a significant advancement in the field of cardiovascular imaging and postoperative management.

The study presented here aligns with a growing body of literature exploring the use of AI and machine learning in medical imaging, particularly in the context of cardiovascular diseases and aortic aneurysms [27,28]. Recent advancements have demonstrated the potential of AI algorithms in automating complex diagnostic tasks, including the identification of pathologies, such as aneurysms, stenosis, and aortic dissections from imaging datasets [29]. However, the application of unsupervised learning techniques, like the VAE used in this study, remains relatively novel in this field, especially in the specific context of post-operative assessment following the FET procedure. The advantage of this kind of algorithm is that it is unsupervised, so the training and verification of the results are all done by the algorithm, and there is no need to manually mark the presence and absence of thrombosis. The main motivation of our study was to demonstrate the proof-of-concept that the implementation of a 2DCNN VAE with the existence of a 2D latent space addresses the delivery of the desirable outcomes as mentioned above. To be trained, VAEs require a large amount of training data, which are obtainable as 2D images reconstructed along the long axis of the aorta, which comprises the region of interest. A 2D latent space was chosen to separate images containing false lumen patency, false lumen thrombosis, and no presence of a false lumen. The MPR images represent axial cross-sections perpendicular to the aortic centerline containing the size-varying lumen of the aorta and the thrombus within the FL. VAEs map the high-dimensional image data into parameters of probability distributions, i.e., the mean and variances of normalized Gaussian distributions. The purpose of this approach is to create a continuous, structured latent space in which subspaces contain different kinds of images. In our study, the encoder 2D CNN approximately defined the so-called posterior distribution and calculated the conditional distribution of the latent representation. The decoder 2D CNN successfully defined the conditional distribution of the observation. The algorithm must find the best encoder/decoder pair to keep the maximum information when encoding and a minimum reconstruction error when decoding. The attribution of a thrombus score was successful, as subspaces in the latent space could be separated into the two classes of interest, i.e., presence versus absence of a thrombus. Validation of the VAE performance was assessed by two clinicians in comparing the 3D curved MPR CTA images versus the visualization of the thrombus score for the slices in each dataset and yielded good agreement. The current study demonstrates the feasibility of using an unsupervised convolutional VAE for automated thrombus formation, which is encouraging compared to supervised CNN-based classifiers, which sometimes rely on time-intensive extensive manual annotations. Direct comparison with other traditional algorithms was not performed, the favorable outcome of our approach however warrants this comparison to find the best approach for this task.

The average thrombus score provides a metric for comparison with other clinical variables to potentially investigate the predictive power of the VAE to aid in clinical decision making. Moreover, the algorithm’s capability to provide patient-specific thrombus scores allows for personalized follow-up strategies, comparing the amount of thrombus over time. This could potentially improve outcomes by identifying patients at higher risk for negative aortic remodeling and possible complications early.

In clinical practice, the application of such an unsupervised AI tool could enhance the efficiency and accuracy of post-operative monitoring. By aligning with current trends in personalized medicine, this study also underscores the potential for AI to contribute to tailored follow-up protocols based on precise, patient-specific data.

### Study Limitation

The emphasis of our study is on the feasibility of integrating an AI algorithm with a potential clinical application, i.e., providing a tool for aiding the clinician in determining the presence of aortic thrombus. Integrating clinical knowledge into the algorithm may help reduce the user interaction time with the CTA image interpretation and may also improve accuracy. While feasibility has been demonstrated, additional investigations are needed to establish the efficacy of this algorithm as well as its limitations.

## 5. Conclusions

The presence of false lumen thrombosis during follow-up examinations after the Frozen Elephant Trunk procedure is a critical indicator of positive aortic remodeling and helps to predict patient outcomes and guide clinical decisions. This study introduces a proof-of-concept for an artificial intelligence algorithm using variational autoencoders to automate the classification and quantification of false lumen thrombosis in CTA imaging. The algorithm accurately distinguished between the presence and absence of a thrombus in the false lumen, providing consistent, patient-specific thrombus scores. By automating this process, it addresses the inefficiencies and variability of manual analysis, offering improved diagnostic accuracy, reduced evaluation times, and enhanced identification of high-risk patients. Integration into clinical workflows could significantly streamline postoperative monitoring and support personalized follow-up protocols. Further studies are warranted to establish the efficacy and limitations of this algorithm and to explore the integration of this AI-based technique into the clinical workflow. The results underscore the potential of artificial intelligence in advancing precision medicine and improving long-term outcomes for patients with complex aortic pathologies.

## Data Availability

The datasets analyzed for this study are available on request to the corresponding author.

## Supplementary Materials

The following supporting information can be downloaded at: https://www.mdpi.com/article/10.3390/diagnostics14242853/s1, Video S1: Training of the VAE algorithm illustrated by the reconstruction of aortic cross-sectional images by the decoder during the learning process. Weights in the convolutional networks are determined by backpropagation for minimizing the root mean error between the reconstructed and the original image and by minimizing the Kullback–Leibler divergence to achieve a smooth (Gaussian) distribution in the latent space.

## Abbreviations

2D CNN	2D convolutional neural networks
AI	artificial intelligence
CTA	computed tomography angiographic
FET	Frozen Elephant Trunk
FL	false lumen
MPR	multi-planar reconstruction
TL	true lumen
VAE	variational autoencodertraditiona
